# Quantification and origin of differential pulmonary blood flow in patients with a Fontan circulation

**DOI:** 10.1186/1532-429X-18-S1-P189

**Published:** 2016-01-27

**Authors:** Senthil Ramamurthy, Sassan Hashemi, Gary R McNeal, Tim Slesnick

**Affiliations:** 1 Cardiovascular Imaging Research Core, Children's Healthcare of Atlanta, Atlanta, GA USA; 2 School of Medicine, Emory University, Atlanta, GA USA; 3 Cardiology, Children's Healthcare of Atlanta, Atlanta, GA USA; 4 Siemens Medical Solutions, Malvern, PA USA

## Background

"Hepatic factor" is found in inferior systemic venous return, and is required by pulmonary tissue to prevent formation of pulmonary arteriovenous malformations (PAVMs). Quantification and origin of differential systemic venous return to branch pulmonary arteries (PAs) in the Fontan population is therefore critical to assess the risk for PAVMs.

## Methods

Patients with a Fontan circulation were prospectively consented to undergo three dimensional phase contrast (4Dflow) imaging during a clinical cardiac MRI evaluation which also included standard two dimensional phase contrast (2D-PC) imaging of all pertinent vessels. Differential pulmonary blood flow was calculated using standard 2D-PC imaging of the right and left pulmonary arteries (RPA and LPA). On the 4Dflow datasets, an expert reader defined cross sections of the RPA, LPA, and the superior and inferior venae cavae (SVC and IVC) within the Fontan circuit using prototype 4Dflow software (Siemens Healthcare, Erlangen, Germany). These defined the analysis and seed planes respectively. Particles were emitted from either the SVC or IVC plane, and quantification was performed at the outlets using the 4Dflow software. The 3D pathlines were then exported for further analysis. Custom software was written in MATLAB (Mathworks, Natick, MA) for flow quantification. The terminal spatial position of each particle was determined. The number of particles that crossed into the RPA and LPA were counted. The differential pulmonary blood flow was calculated for each simulation as the ratio of particles in the RPA to LPA. Internal consistency of the 4Dflow measurements was validated using conservation of mass analysis. Systemic venous flow by origin (IVC or SVC) and total systemic venous flow into each branch PA were calculated. Preferential flow was defined as flow > 70% into one of the branch PAs.

## Results

**Figure 1:**
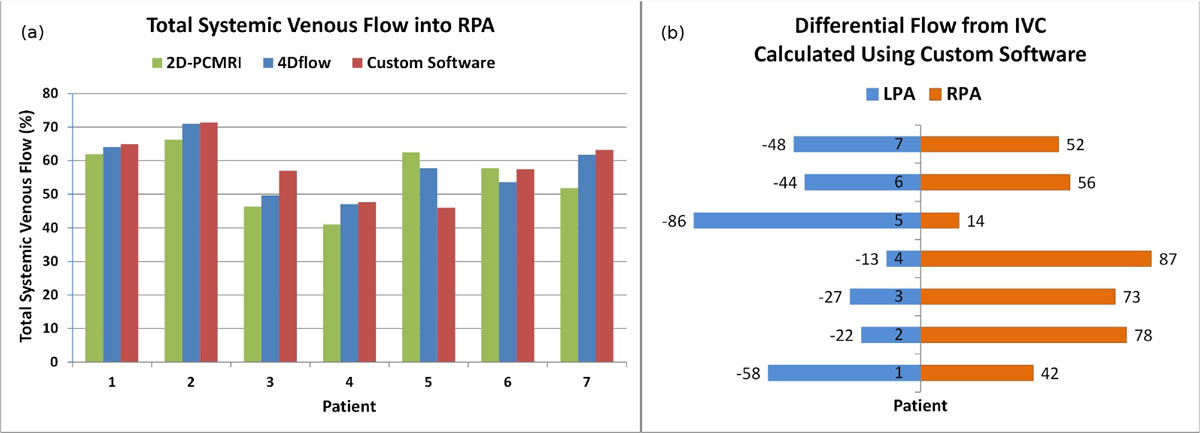
Figure 1

A total of 7 patients (5 male) were enrolled between November, 2013 and March, 2015. The mean age was 10.4 ± 3.1 years. The age at Fontan surgery was 2. 8 ± 1.4 years. The custom MATLAB software successfully analyzed differential pulmonary flow from both the SVC and IVC individually, as well as in combination. There was strong agreement (ICC=0.70) in total systemic venous flow into the RPA by 2D-PCMRI, prototype 4Dflow software and the custom technique. Figure 1(a) demonstrates there were no patients where overall systemic venous flow was preferential into the RPA on any platform. However, 3 patients (#2, #3 and #4) had preferential flow from the IVC into the RPA and 1 patient (#5) had preferential flow from the IVC into the LPA as shown in Figure 1(b) when calculated using the custom software.

## Conclusions

We have developed a novel technique to assess the origin of differential pulmonary blood flow from 4Dflow MRI. This technique may help in identifying patients at risk for developing PAVMs, including those who may have symmetric total systemic venous flow into the branch PAs, but preferential flow from the IVC to one branch pulmonary artery.

